# Attenuation model of tunnel blast vibration velocity based on the influence of free surface

**DOI:** 10.1038/s41598-021-00640-9

**Published:** 2021-10-26

**Authors:** Baoxin Jia, Linli Zhou, Jiaojiao Cui, Hao Chen

**Affiliations:** School of Civil Engineering, Liaoning Technical University, Fuxin, 123000 Liaoning China

**Keywords:** Civil engineering, Petrology, Seismology

## Abstract

In tunnel blasting excavation, it is important to clarify the attenuation law of blast wave propagation and predict the blast vibration velocity effectively to ensure safe tunnel construction and protection design. The effects of the free surface area its quantity on the blast vibration velocity are considered, and free surface parameters are introduced to improve the existing blast vibration velocity prediction formula. Based on the Tianhuan railway Daqianshiling tunnel project, field blast vibration monitoring tests are performed to determine changes in the peak blasting vibration velocity based on the blast distance and free surface area. LS-DYNA is used to establish tunnel blasting excavation models under three operating conditions; subsequently, the attenuation law of blast vibration velocity and changes in the vibration response spectrum are analysed. Results show that the free surface area and number of free surfaces enable the blast vibration velocity to be predicted under various operating conditions: a smaller free surface area results in a narrower frequency band range, whereas more free surfaces result in a narrower frequency band range. The improved blast vibration velocity prediction formula is validated using field and numerical test data. It is indicated that the improved formula is applicable to various tunnelling conditions.

## Introduction

Blasting technology is vital to transportation, construction, water conservancy, and mining industries; however, in tunnel blasting excavation, the dynamic effects caused by blast vibration can be detrimental to the surrounding rock environment and lining structure. To avoid the damage and effect caused by blast vibration, the investigation into the blast vibration attenuation mechanism has received significant attention, among which the prediction and control of blast vibration intensity are crucial. The peak particle vibration velocity is representative of the blast vibration intensity, and the best criterion for estimating the vibration damage level of structures is the blast vibration velocity. Therefore, the peak particle vibration velocity is used as the control criterion for blast vibration^1^. Accurately predicting the blast-induced particle vibration velocity to ensure the safe and smooth operation of blasting projects is the main method for controlling blast vibration hazards^2–5^.

Studies pertaining to the blast vibration velocity as an object have been conducted extensively. For example, Cao et al.^4^ analysed the transmission propagation law of blast stress waves and solved the safety threshold of blast vibration velocity based on the ultimate strength theory. Sun et al.^6^ proposed a value of blast vibration velocity for shallow rock, and Singh et al.^7^ determined the safety level of the ground structure under blast vibration. Both discussed the problem of determining the safety value of blast vibration from the blast vibration velocity. Zhuang et al.^8^ monitored the blast vibration law of semi-coal rock laneway, obtained the blasting main vibration frequency from 50 to 250 Hz, and performed Sadoff formula regression fitting to calculate the safe allowable distance for blasting in different rock layers. Kim et al.^9^ proposed geometric damping coefficients; Tian et al.^10^ considered different paths of vibration propagation under laminated rock conditions and investigated the blast vibration propagation and attenuation laws. Yu et al.^11^ conducted field tests to investigate the propagation law of blast vibration in the surface and blast-face side of shallow buried small clearance tunnels. Zhu et al.^12^ preferred a blast prediction model that considered the blasting charge and blast centre distance, as well as one that considered the blasting charge and height difference; subsequently, they established a segmental prediction model. Wu et al.^13^ elucidated the amplification effect of step terrains on the blast vibration velocity and established a vibration velocity prediction model for step terrains. Zhu et al.^14^ established a multi-degree-of-freedom model of blast vibration and its differential equations of motion to propose a method for predicting the blast vibration velocity of a multihole cut blast in layered rock masses. Applying computer theory technology, Feng^15^ used empirical formulas and a grey model to predict the peak vibration velocity. Zou et al.^16^ used asymmetric triangular fuzzy numbers, which were used to determine the parameters of a prediction formula, whereas Ma et al.^17^, Singh et al.^18^, and Wei et al.^19^ used neural network algorithms, artificial intelligence, and finite element simulation techniques to establish prediction models, respectively. Yue et al.^20^ combined the particle swarm algorithm and least-squares support vector machine model to predict the peak blast vibration velocity. Cai et al.^5^ improved the Elman neural network model by considering the free surface area to predict the blast vibration velocity using the beetle antenna search algorithm.

Whereas the above mentioned studies considered many factors such as total charge, blast distance, elevation, hole diameter, number of holes, and blasting duration to establish prediction models including the neural network model and least squares support vector machine, less research has been conducted regarding the effect of free surface parameters on blast vibration attenuation. In addition, in tunnel blasting excavation, the cutting holes create a free surface, and the area and number of free surfaces affect blasting excavation and tunnel blast vibration attenuation. In this study, based on the Sadoff empirical formula and the vibration velocity attenuation formula of Lu et al.^21^, free surface parameters are introduced into a formula to improve the prediction of the peak blast vibration velocity.

## Tunnel blast vibration velocity prediction formula

In the blasting industry, blast vibration hazard effects are evaluated by predicting the peak particle vibration velocity, and blast vibration control measures are formulated; however, the prediction accuracy is a longstanding issue in the blasting industry. Based on extensive engineering experience and research, scholars have proposed different empirical formulas for the blast vibration velocity. Currently, the empirical formula of the peak blast vibration velocity proposed by Sadoff, which is shown in Eq. (1), is the most widely used in China’s blasting industry:

1 $$V = K\left( {\frac{{Q^{{{1 \mathord{\left/ {\vphantom {1 3}} \right. \kern-\nulldelimiterspace} 3}}} }}{R}} \right)^{q}$$

where *V* is the peak blast vibration velocity (cm/s); *Q* is the maximum single detonation charge (kg); *R* is the source distance (m); *K* and *q* are field coefficients.

The formula above does not reflect the effects of explosive properties, explosive type, charging method, hole size, and surrounding rock parameters on the blast vibration velocity. Lu et al. obtained an equation expressing particle blast vibration velocity using the fluctuation method to reflect the above mentioned factors. Based on the wavelet of the long column charge and column surface wave theory, the Helen solution for the stress wave field excited by the column charge package was analysed to derive an equation for the peak blast vibration velocity^21^, which is shown in Eq. (2).

2 $$V = k\frac{{p_{0} }}{{\rho c_{p} }}\left( \frac{b}{R} \right)^{\alpha }$$

where *k* is the improvement factor associated with the blasting conditions, *p*_0_ the initial pressure of the blast-generated gas in the hole (Pa), *b* the radius of the hole (mm), *ρ* the rock density (kg/m^3^), *R* the distance of the detonation point from the particle (m), *c*_*p*_ the rock longitudinal wave velocity (m/s), and *α* the blast vibration attenuation index. When the charge is coupled, *p*_0_ = *p*_*e*_, where *p*_*e*_ = *ρ*_*e*_*D*^2^/2(*γ* + 1). When the charge is uncoupled, if the uncoupled factor *b*/*a* is smaller, then *p*_0_ = *p*_*e*_(*a*/*b*)^2*γ*^; if *b*/*a* is larger, then $p_0=[{\rho_{e}}{D^{2}}/2 (\gamma+1)]^{\gamma_0/\gamma}{p_{k}}^{({\gamma-\gamma_{0}})}(a/b)^{2\gamma_0}$, where *ρ*_*e*_ is the explosive density, *D* the explosive detonation velocity, *γ* the adiabatic index, *p*_*k*_ the critical pressure of the explosive, *p*_*e*_ the average explosive detonation pressure, and *a* the radius of the charge.

Generally, the surface of a blasted rock in contact with air is referred to as the free surface. The larger the free surface area and the greater the number of free surfaces, the more prominent is the blasting effect. The free surface is one of the important factors affecting the blasting effect. Hence, the prediction accuracy can be improved by investigating the effects of free surface factors on the blast vibration velocity and the influence law of the free surface into the prediction formula.

The free surface parameters were introduced into the vibration velocity calculation formula to improve the peak vibration velocity prediction formula based on the Sadoff formula and the peak vibration decay formula by Lu et al. This is because the larger the free surface area, the greater is the number of free surfaces, and the smaller is the surrounding blast vibration^22^. Therefore, the free surface area *S*, number of free surfaces *m*, and free surface index *β* were selected as the free surface parameters and then incorporated into Sadoff’s formula [as shown in Eq. (1)] and Lu et al.’s formula [as show in Eq. (2)]. The inconsistency in the magnitude after considering the direct addition of *S* was considered by incorporating the term (*S*/*mR*^2^)^*β*^; hence, the improved formulas are as shown in Eqs. (3) and (4).

3 $$V = K\left( {\frac{{Q^{{{1 \mathord{\left/ {\vphantom {1 3}} \right. \kern-\nulldelimiterspace} 3}}} }}{R}} \right)^{q} \left( {\frac{S}{{mR^{2} }}} \right)^{\beta }$$

4 $$V = k\frac{{p_{0} }}{{\rho c_{p} }}\left( \frac{b}{R} \right)^{\alpha } \left( {\frac{S}{{mR^{2} }}} \right)^{\beta }$$

where *S* is the free surface area, which is the surface area where the hole arrangement is located; *m* is the number of free surfaces, which is the number of blasting rock mass surfaces exposed to air; *β* is the free surface index. The remaining variables are the same as those in Eqs. (1) and (2).

## Tunnel blasting field tests

### Field overview

**Figure 1:**
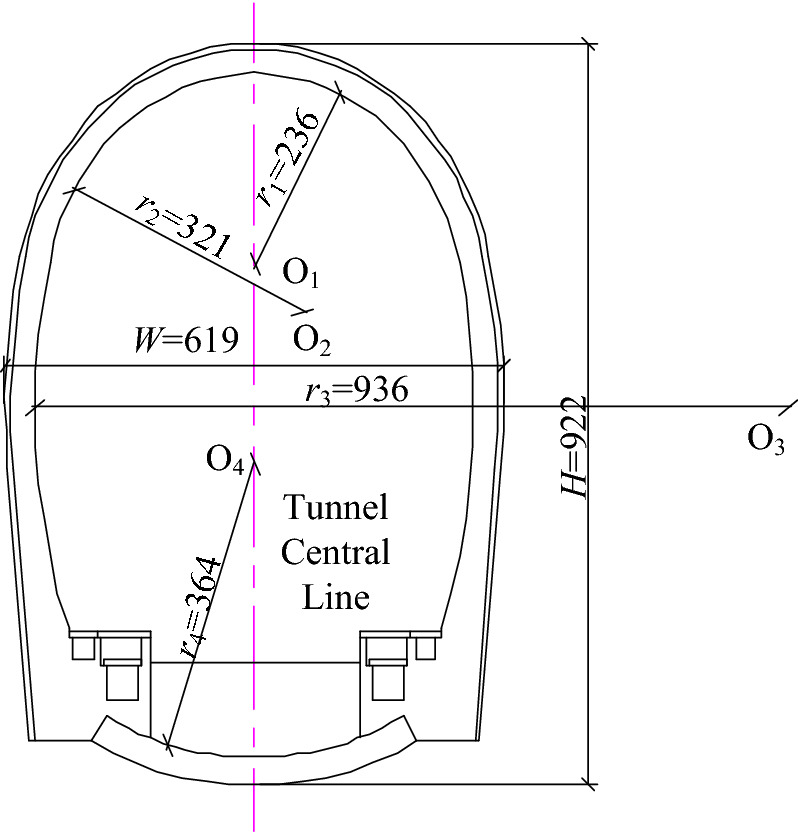
Tunnel cross-section dimensions (units: cm).

Based on the Tianhuan railway Daqianshiling tunnel as an example, the total length of the tunnel was 2460 m, which reflects a medium-sized tunnel in a railway tunnel. The tunnel inlet and outlet sections DK69 + 225–DK69 + 495 and DK71 + 180–DK71 + 685 contained level IV–V surrounding rocks, excavated via the overrunning small conduit pre-supporting step method, and the middle section DK69 + 495–DK71 + 180 contained level II–III surrounding rocks, excavated via the full section method; both excavation methods are the blasting excavation method. Based on the geological survey data of the project and indoor rock mechanics test, it was discovered that the surrounding rock of the tunnel was quartz sandstone, which is a hard rock, and the surrounding rock parameters are listed in Table 1. The tunnel cycle feed was set as 1.6 m. The length of the steps was 10 m, which is considered short. The tunnel cross-section was a horseshoe-shaped four-centred circular arch tunnel, and the tunnel cross-section dimensions are shown in Fig. 1.

**Table 1:** Surrounding rock physics parameters.

Density (kg/m^3^)	Rock modulus (Pa)	Tangential modulus (Pa)	Poisson’s ratio	Hardening parameters	Yield stress (Pa)	P wave velocity (m/s)	Cohesion (Pa)	Angle of friction
2430	4.83 × 10^9^	1.13 × 10^8^	0.26	0.5	3.0 × 10^7^	1560	5 × 10^6^	30°

**Figure 2:**
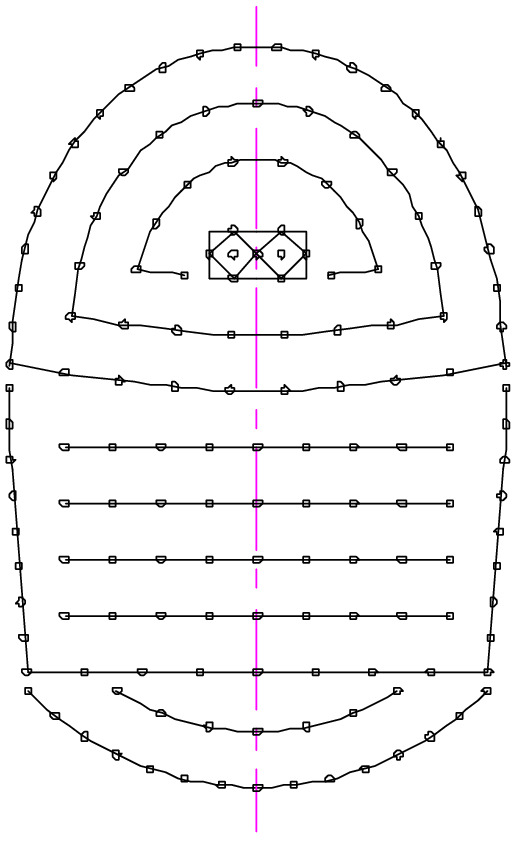
Blast hole layout.

The blasting excavation was performed using rock emulsion explosives with a TNT equivalent value of 0.73^23^. The explosive density was 1200 kg/m^3^, the explosive detonation velocity was 4500 m/s, and the adiabatic index was 3. In the blasting design, 153 holes were arranged in the outer contour of the tunnel, including two bum holes, seven cutting holes, 87 reliever holes, and 57 rim holes; the hole arrangement is shown in Fig. 2. The diameter of the hole was 35 mm, and the charging method was coupled. The total amount of charge in the entire section was 152.16 kg, 72.48 kg in the upper step and 79.68 kg in the lower step.

### Tunnel blast vibration monitoring

**Figure 3:**
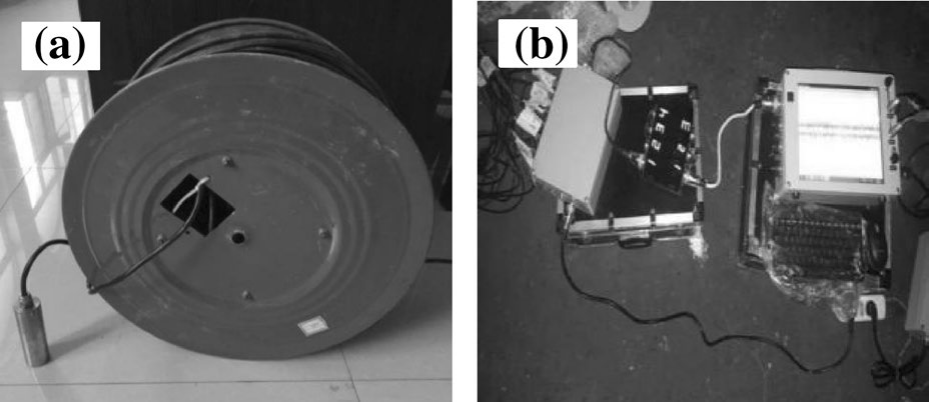
Data acquisition system: (**a**) Sensors and cables. (**b**) Data hubs, converters, and signal collectors.

The data acquisition system developed by the Institute of Geology, China Earthquake Administration, was used for the blast vibration monitoring test. It comprised five parts: sensors, cables, data hubs, converters, and signal collectors (Fig. 3). The signal acquisition frequency of the blast test was set to 50 kHz, and the sensor was a non-directional velocity sensor.

Seven monitoring points were established for blast vibration monitoring, and five blasting tests were conducted. Owing to the complexity of the field environment and the difference in the blasting effect of the rim holes, the free surface area of each blast changed, and the free surface areas of the fourth and fifth blasts increased because of the addition of an emergency avoidance hole in those areas. The monitoring points were fixed to the right side of the interior of the tunnel. The monitoring points were named using two digits, where the first digit represents the serial number of the blast, and the second digit represents the location of the monitoring point. For example, 3–2 indicates the third blast-monitoring point, No. 2. The peak vibration velocity of the extracted monitoring signal, the area of the free surface of each blast, and the distance of each monitoring point from the blast free surface are listed in Table 2.

**Table 2:** Site blasting vibration velocity monitoring data.

First blast	Monitoring points	1–1	1–2	1–3	1–4	1–5	1–6	1–7	Free surface area (m^2^) 51.03
	Blast distance (m)	42	55	68	80	95	120	135	
	Peak vibration velocity (cm/s)	7.847	6.415	4.980	4.077	2.977	2.621	1.350	
Second blast	Monitoring points	2–1	2–2	2–3	2–4	2–5	2–6	2–7	Free surface area (m^2^) 52.21
	Blast distance (m)	45	58	71	83	98	123	138	
	Peak vibration velocity (cm/s)	7.275	4.819	3.749	3.118	2.727	2.500	1.290	
Third blast	Monitoring points	3–1	3–2	3–3	3–4	3–5	3–6	3–7	Free surface area (m^2^) 51.47
	Blast distance (m)	48	61	74	86	101	126	141	
	Peak vibration velocity (cm/s)	6.330	4.551	3.707	2.701	2.653	2.356	1.123	
Fourth blast	Monitoring points	4–1	4–2	4–3	4–4	4–5	4–6	4–7	Free surface area (m^2^) 58.15
	Blast distance (m)	66	79	92	104	119	144	159	
	Peak vibration velocity (cm/s)	4.556	2.459	2.616	1.675	1.585	1.169	0.611	
Fifth blast	Monitoring points	5–1	5–2	5–3	5–4	5–5	5–6	5–7	Free surface area (m^2^) 58.24
	Blast distance (m)	69	82	95	107	122	147	162	
	Peak vibration velocity (cm/s)	4.071	3.210	2.259	1.934	1.250	0.801	0.362	

### Comparison of blast velocity prediction formulas

**Figure 4:**
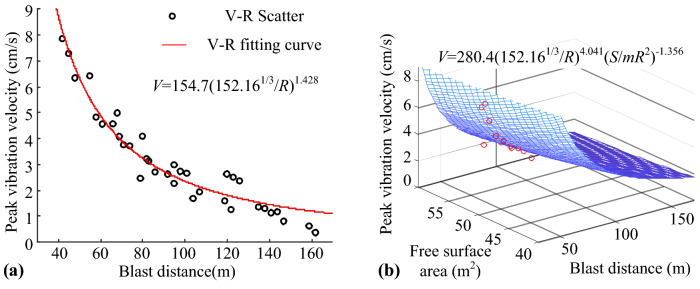
Fitting curve of site test: (**a**) Base formula fitting. (**b**) Improved formula fitting.

The burst source distance *R*, peak vibration velocity *V*, and free surface area *S* listed in Table 2 were used to establish the elements individually in the corresponding column vector, based on the prediction formula shown in Eqs. (1) to (4). The MATLAB Curve Fitting Tool was used for fitting, and the fitting curve is shown in Fig. 4, whereas the fitting parameters are listed in Table 3. It was clear that the Sadoff formula and Lu et al. formula for predicting the peak vibration velocity considering the blast vibration parameters were different. However, the relationship between the change in the peak vibration velocity and the propagation distance was the same; hence, the effects of the two fittings were the same.

**Table 3:** Field test fitting parameters.

Formula number	(1)	(3)	(2)	(4)
Best estimation of parameter	*K* = 154.7	*K* = 280.4	*k* = 0.1922	*k* = 0.3456
	*q* = 1.428	*q* = 4.041	*α* = 1.428	*α* = 4.041
		*β* = − 1.356		*β* = − 1.356
SSE	7.914	6.515	7.914	6.515
RMSE	0.4897	0.4512	0.4897	0.4512
R-square	0.9340	0.9456	0.9340	0.9456
Adjusted R-square	0.9320	0.9422	0.9320	0.9422

The summed variance (SSE), standard deviation (RMSE), coefficient of determination (R-square) and adjusted coefficient of determination (adjusted R-square) in Table 3 were calculated and generated using the MATLAB Curve Fitting Tool. They were calculated as shown in Eqs. (5)–(8), respectively, as follows:

5 $${\text{SSE}} = \sum\limits_{i = 1}^{n} {w_{i} \left( {y_{i} - \hat{y}_{i} } \right)^{2} }$$

6 $${\text{RMSE}} = \sqrt {\frac{1}{n}\sum\limits_{i = 1}^{n} {w_{i} \left( {y_{i} - \hat{y}_{i} } \right)^{2} } }$$

7 $${\text{R - square}} = 1 - \frac{{\sum\nolimits_{i = 1}^{n} {w_{i} \left( {y_{i} - \hat{y}_{i} } \right)^{2} } }}{{\sum\nolimits_{i = 1}^{n} {w_{i} \left( {y_{i} - \overline{y}_{i} } \right)^{2} } }}$$

8 $${\text{Adjusted R - square}} = 1 - \frac{{\left( {1 - R^{2} } \right)\left( {n - 1} \right)}}{n - p - 1}$$

In the equations above, *w*_*i*_ is the data sample weight, *y*_*i*_ the original data, $\hat{y}_{i}$ the predicted data, $\overline{y}_{i}$ the original data mean, *n* the number of samples, and *p* the number of features.

The closer the SSE and RMSE are to 0, the closer are the R-square and adjusted R-square to 1. These values reflects a well fit model, indicating that the independent variables can better reflect the dependent variable. Therefore, these four fitting parameters can be used to compare the strengths and weaknesses of the fit of the prediction formulas. By comparing the data shown in Table 3, it can be concluded that the blast vibration velocity prediction formula with the addition of the free surface parameters is better in terms of fitting. The Fig. 4 shows that the distribution of scattered points on the curve is more scattered than the distribution of scattered points on the surface. This indicates that the effects of the free surface area and number of free surfaces on the peak blast vibration velocity are non-negligible. Therefore, free surface parameters must be incorporated into the base prediction formula.

## Tunnel blasting numerical tests

Owing to certain limitations of the field tests, e.g. small variations in the free surface area, vibration velocity monitoring was performed only during the full section stage of tunnel excavation. Therefore, the effects of the free surface parameters of tunnel blasting on the blast vibration velocity is discussed based on numerical calculations.

### LS-DYNA computational modelling

**Figure 5:**
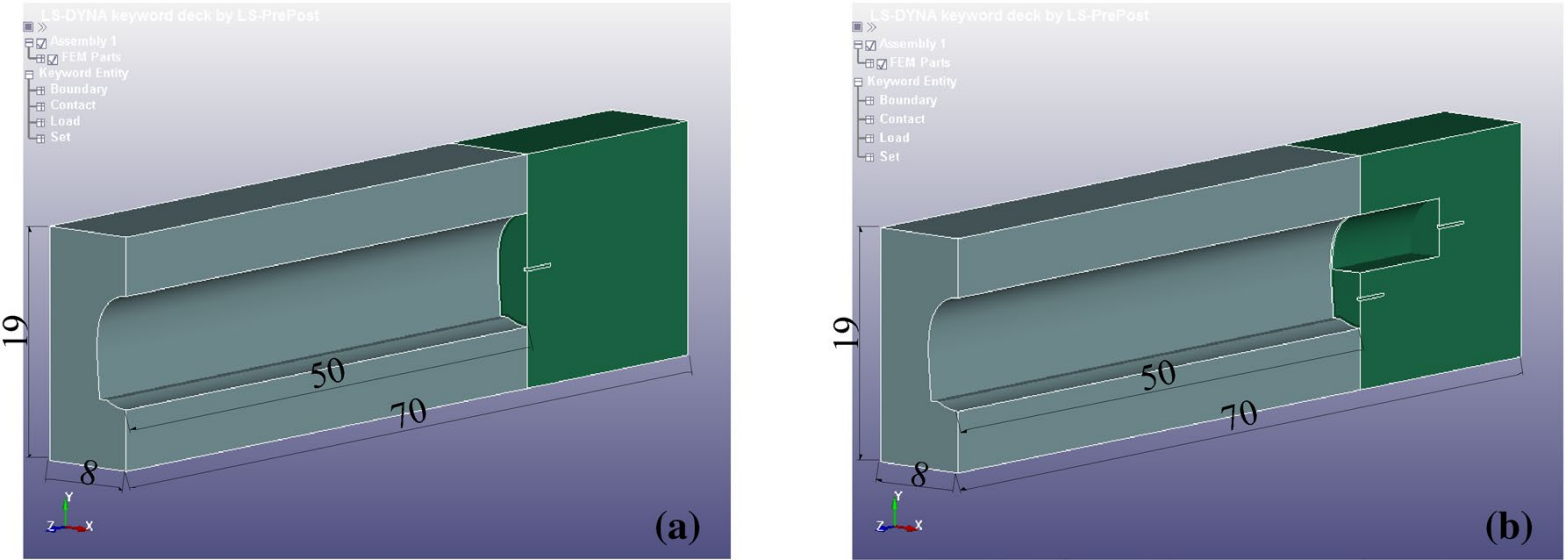
Tunnel numerical model: (**a**) Full section model. (**b**) Step model.

LS-DYNA analysis software was used to establish the tunnel model based on the operating conditions of the Daqianshiling tunnel. Because the tunnel section is symmetrical on the left and right, to improve the software calculation efficiency, the model was established using the left half, where a symmetric boundary condition was set in the symmetric section. The calculated model was 70 m long (z-direction), 8 m wide (x-direction), and 19 m high (y-direction), and the established model is shown in Fig. 5. Two numerical models were established: a full section model (as shown in Fig. 5a) and a step model (as shown in Fig. 5b).

Each model was segregated into two modules, one for the excavated section, and the other for the unexcavated section. Furthermore, the same physical and mechanical parameters were set for the surrounding rock in both sections. In the full section model, the length of the excavated section was 50 m, i.e. the depth of the tunnel was 50 m. In the step model, the length of the step was 10 m, and the remaining dimensions were the same as those of the full section model. The model grid size was 0.25 m. The hole was located in the centre of the palm face, occupying a cell with a depth of 3 m, and 12 cells were removed. Because the model boundary was limited, i.e. much smaller than the field, when the fixed boundary conditions are used, the reflection of the boundary on the blast wave is evident; therefore, the boundary conditions were set to the no-reflection boundary, ‘BOUNDARY_NON_REFLECTING’, to reduce the reflected wave generated by the model boundary to the model perturbation. The tunnel calculation model was segregated into three regions as follows: (1) Full section blasting—a full section area measuring 48.9515 m^2^, a free surface is present, and the source is located in the centre of the full section; (2) upper step blasting—an upper step area measuring 22.8886 m^2^, a free surface is present, and the source is located in the centre of the upper step section; (3) lower step blasting—a lower step area measuring 26.0629 m^2^, two free surfaces are present, and the source is located in the centre of the lower step section.

The Mohr–Coulomb material keyword, ‘MAT_MOHR_COULOMB’, was selected for the surrounding rock constitutive model. The blast load was set using the ‘LOAD_BLAST_ENHANCED’ function^24^, which simulates the action of the blast wave with the geotechnical mass based on ‘LOAD_BLAST_SEGMENT_SET’. This blast setting method disregards the shape of the explosive charge and the propagation of the blast shock wave in air; as such, the charge and air need not be modelled, which reduces computational time. Zhang et al.^25^ and Gilson et al.^26^ applied the method above and verified its feasibility.

In this method of applying blast loads, the TNT equivalent of the explosive must be defined; additionally, the blast action plane and blast source coordinates must be selected. The blast source coordinates were set as the centre coordinates of the hole (see Table 4), and the blast action plane was selected as the inner side of the hole. Because the cell measured 0.25 m and the actual hole diameter was 0.035 m, the area of one cell corresponded to 65 actual holes after conversion. Because the average charge of a hole was 1 kg (based on the engineering blasting design), 65 kg of explosives were set in the holes of all three types of models; furthermore, it was ensured that the explosives would not exceed the maximum charge under various operating conditions. The emulsified explosive charge was converted to a TNT equivalent of 47.45 kg. The blasting parameters of the three types of models were unified and satisfied the requirements of the comparison test.

**Table 4:** Blast source coordinates (units: m)

Blast source	*x*_0_	*y*_0_	*z*_0_
Full section	8.00000	9.60910	48.2716
Upper step	8.00000	11.8502	38.3951
Lower step	8.00000	7.36643	48.2716

**Figure 6:**
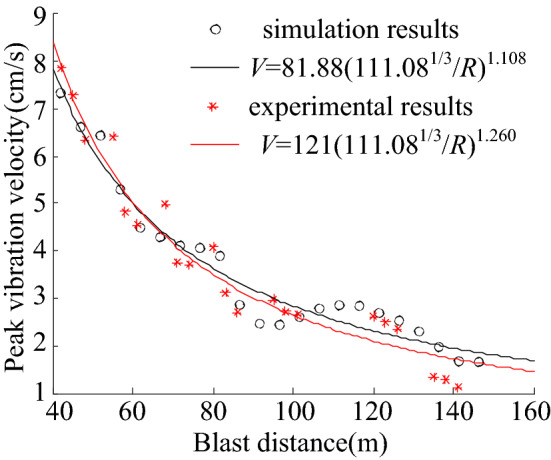
Validation of numerical model.

To verify that the model above can reflect the field test results, the length of the excavated section of the model was extended to 150 m, which eases comparison with field monitoring data. Because the field data were only available for the full section excavation stage, model validation was performed only for the full section model. Disregarding the change in the free surface area in the field test, the monitoring point vibration velocity of the first three blasting full sections was selected for the calibration data. The numerical model explosive quantity was adjusted to 111.08 kg TNT equivalent for calculation. A comparison between the numerical simulation and monitoring results is shown in Fig. 6, which shows that the numerical model established in this study can simulate the field test accurately.

### Particle vibration velocity monitoring

**Figure 7:**
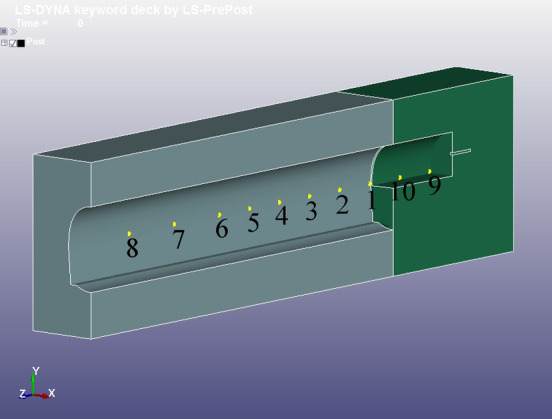
Illustration of monitoring point locations.

The monitoring points were set at the side walls of the upper and lower step separation interfaces and arranged horizontally along the tunnel axis. The monitoring points for the three types of model were in the same position. Eight monitoring points were set in the full section model and the lower step model, beginning from the palm face; the first six monitoring points were spaced 5 m apart (20 units), and the last two monitoring points were spaced 7.5 m apart (30 units). In the upper step model, the eight monitoring points were retained, and two monitoring points were added on the same horizontal line on the step with an interval of 5 m (20 units) (see Fig. 7). The coordinates of each monitoring point are listed in Table 5, and the monitoring points were numbered based on the node number. The resultant velocity of the monitoring points was extracted via numerical model calculations. The effects of the free surface parameters on the blast vibration velocity were compared and analysed.

**Table 5:** Monitoring point coordinates (units: m).

Serial no.	Monitoring point no.	*x*_*i*_	*y*_*i*_	*z*_*i*_	Remarks
1	2341761	4.90407	9.66302	50.2488	The monitoring points numbered from 1 to 8 are the monitoring points common to the three models, and the numbering increases from the smallest to the largest as the distance from the palm surface increases. The monitoring points numbered 9 and 10 are the additional monitoring points on the upper platform, and the numbering increases from the smallest to the largest as the distance increases from the upper platform palm surface
2	2341741	4.90407	9.66344	55.2239	
3	2341721	4.90407	9.66344	60.1990	
4	2341701	4.90407	9.66344	65.1741	
5	2341681	4.90407	9.66344	70.1492	
6	2341661	4.90407	9.66344	75.1244	
7	2341631	4.90407	9.66344	82.5871	
8	2341601	4.90407	9.66344	90.0498	
9	2887082	4.95309	9.66237	40.3704	
10	2887062	4.95292	9.66239	45.3086	

**Figure 8:**
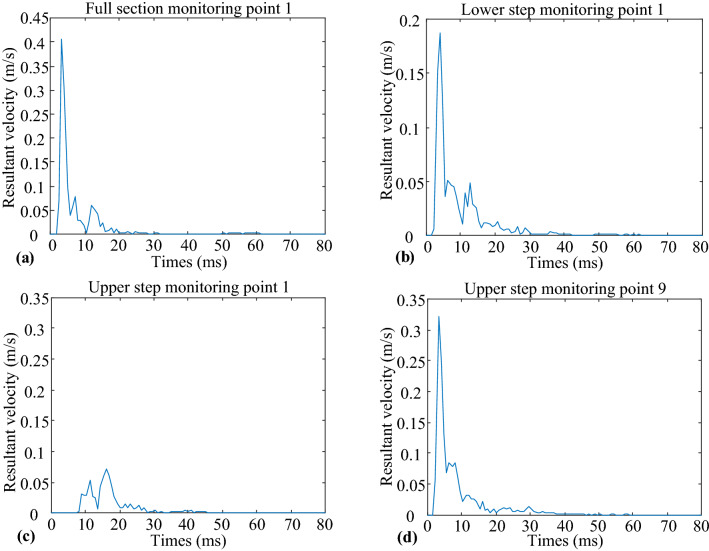
Time course curve of resultant velocity of blast vibration: (**a**) Full section monitoring point 1. (**b**) Lower step monitoring point 1. (**c**) Upper step monitoring point 1. (**d**) Upper step monitoring point 9.

Each monitoring point blast distance can be determined using the blast source coordinates and monitoring point coordinates, and the blast distances are shown in Table 6. The velocity time course curves are shown in Fig. 8, and the vibration waveforms with monitoring point serial No. 1 in the three models and monitoring point serial No. 9 in the upper step model are listed.

**Table 6:** Calculation of blast distance at monitoring point (units: m).

Serial No.	Full section model	Upper step model	Lower step model
1	3.67383	12.4450	4.33225
2	7.61066	17.2504	7.94956
3	12.3228	22.1309	12.5349
4	17.1838	27.0459	17.3365
5	22.0956	31.9795	22.2146
6	27.0307	36.9244	27.1281
7	34.4549	44.3543	34.5314
8	41.8928	51.7936	41.9557
9	–	4.23935	–
10	–	7.86560	–

**Figure 9:**
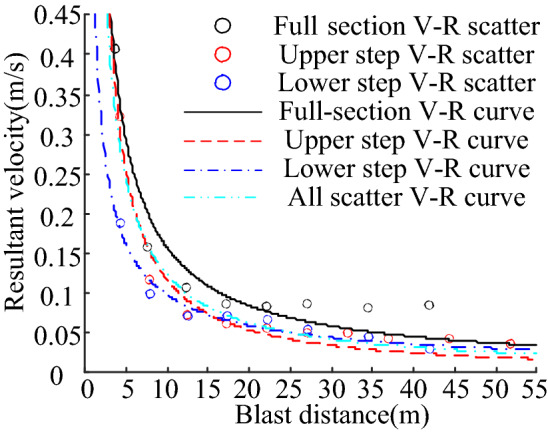
V–R scatter plot and fitting curve.

The peak particle vibration velocity at each monitoring point was determined, as shown in Table 7, and the V–R scatter diagram is plotted in Fig. 9.

**Table 7:** Statistics of peak vibration velocity of each monitoring point (units: m/s).

Serial No.	Full section model	Upper step model	Lower step model
1	0.4073	0.0721	0.1878
2	0.1579	0.0613	0.0987
3	0.1067	0.0555	0.0706
4	0.0859	0.0494	0.0709
5	0.0829	0.0493	0.0664
6	0.0862	0.0424	0.0537
7	0.0811	0.0423	0.0445
8	0.0847	0.0358	0.0293
9	–	0.3221	–
10	–	0.1166	–

### Analysis of peak particle vibration velocity variation

#### Attenuation of peak particle vibration velocity

The MATLAB Curve Fitting Tool was used to fit the scatter data of the three models using Eq. (1) while the full scatter was fitted, and the statistics of the fitted parameters are listed in Table 8.

**Table 8:** Model fitting parameters.

Fitted objects	Full section scatter	Upper step scatter	Lower step scatter	Full scatter
*K* best estimate	0.3933	0.3666	0.2045	0.3338
*q* best estimate	0.904	1.13	0.7301	0.9832
SSE	0.00603	0.003399	0.0007573	0.03037
RMSE	0.0317	0.02061	0.01123	0.03557
R-square	0.9318	0.9496	0.9549	0.8398
Adjusted R-square	0.9204	0.9433	0.9474	0.8331

**Figure 10:**
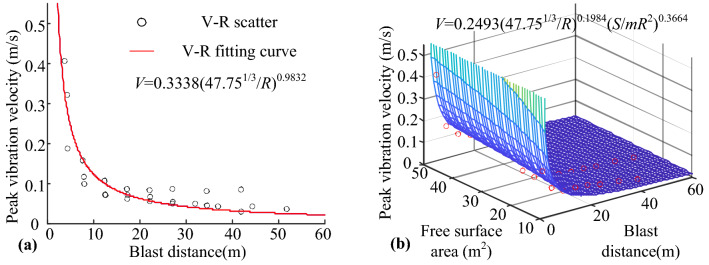
Numerical test fitting curve: (**a**) Base formula fitting. (**b**) Improved formula fitting.

As shown in Table 8, the fitting effect was favourable when fitting the blast vibration decay curves individually; however, the differences among the fitted curves were significant. When all the data were fitted together, the fit was inferior. This indicates that the change in the blasting free surface parameters significantly affected the tunnel blast vibration velocity decay. The results of the numerical calculations were fitted using Eqs. (1) and (4). The fitted curves are shown in Fig. 10, and the fitted parameters are listed in Table 9.

**Table 9:** Numerical test fitting parameters.

Formula number	(1)	(3)	(2)	(4)
Best estimation of parameter	*K* = 0.3338	*K* = 0.2493	*k* = 6.147 × 10^–4^	*k* = 3.187 × 10^–4^
	*q* = 0.9832	*q* = 0.1984	*α* = 0.9834	*α* = 0.1985
		*β* = 0.3664		*β* = 0.3664
SSE	0.03037	0.01371	0.03037	0.01371
RMSE	0.03557	0.02441	0.03557	0.02441
R-square	0.8398	0.9277	0.8398	0.9277
Adjusted R-square	0.8331	0.9214	0.8331	0.9214

The numerical test fitting indexes indicate that the blast vibration velocity prediction formula considering the free surface parameters significantly improved the fitting indexes and yielded a better fitting effect than the base formula. This indicates that Eqs. (3) and (4) can be more widely used for predicting the peak vibration velocity in various tunnel blasting sections.

#### Vibration response spectrum characteristics

**Figure 11:**
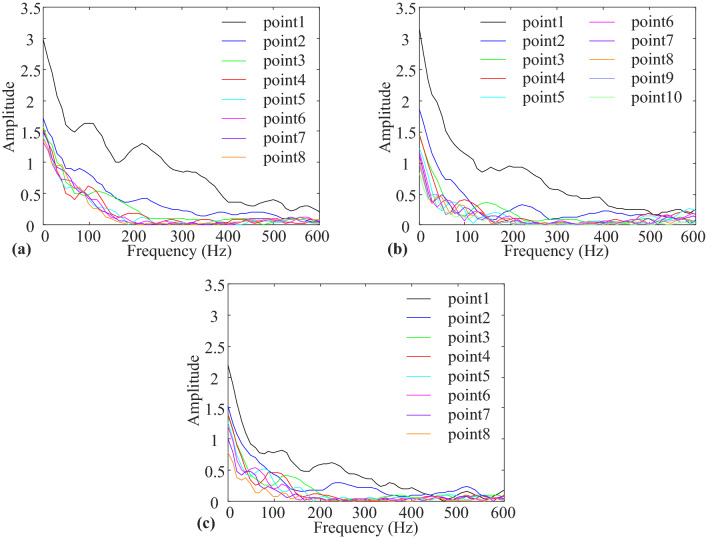
Spectrogram of particle vibration velocity waveform: (**a**) Full section model. (**b**) Upper step model. (**c**) Lower step model.

A spectral analysis of the vibration velocity waveforms at each monitoring point was performed, and the spectrum plots of each monitoring point of the three models were obtained using Fourier transform, as shown in Fig. 11.

The vertical axis amplitude was increased by 100 times, and the frequency range with an amplitude greater than 0.5 was regarded as the main frequency range of vibration. As shown in Fig. 11, the numerical model blast vibration was dominated by low-frequency vibrations. Near the free surface monitoring point, the vibration band was the widest, and the vibration response was the most significant. As the propagation distance increased, the vibration response weakened, and the amplitude decay and band range reduction stabilised. Figure 11a shows that the vibration frequency range of the full section model was primarily in the range of 0–400 Hz, and that the high-frequency components above 150 Hz decayed the fastest. Figure 11b shows that the vibration frequency range of the upper step model was primarily in the range of 0–350 Hz, and the frequency components above 90 Hz decayed rapidly. Figure 11c shows that the overall vibration amplitude of the lower step model was significantly lower than that of the first two models, and that the vibration frequency range reduced significantly reduced, primarily in the range of 0–280 Hz, with the frequency components above 90 Hz decaying rapidly. As shown in Fig. 11, the amplitude of the vibration speed waveform decreased gradually from low frequency to high frequency. The inflexion point of the amplitude of the vibration speed waveform with frequency was at 100 Hz. The amplitude decay rate was high, and the curve in the 0–100 Hz band was steep. After 100 Hz, the amplitude decay rate was low, and the curve was flat. Therefore, with 100 Hz as the cutoff point, the waveform amplitude percentage was calculated for 0–100 Hz, as shown in Table 10. As shown, as the propagation distance increased, the low-frequency component of the waveform at 0–100 Hz increased proportionally and dominated. All the results show that the vibration response from blasting contained both high- and low-frequency components; however, as the blast wave propagated, the high-frequency components decayed rapidly, and the vibration response retained only the low-frequency components and remained in a stable frequency band range.

**Table 10:** Amplitude ratio statistics in 0–100 Hz frequency domain (%).

Serial no	Full section model	Upper step model	Lower step model
9	–	39.34	–
10	–	50.05	–
1	33.24	49.26	39.87
2	44.12	49.27	47.07
3	50.61	49.11	47.92
4	57.32	49.17	54.70
5	64.59	52.40	58.57
6	64.45	56.60	62.48
7	68.64	59.87	61.14
8	70.57	64.78	60.55

Comparing the three numerical models, it was discovered that the widths of the initial frequency bands differed for different free surface areas and numbers. It was observed that a smaller free surface resulted in a narrower frequency band range, whereas more free surfaces resulted in a narrower frequency band range. The step model filtered out more high-frequency components and retained fewer low-frequency components.

## Discussion and conclusions

In previous studies, the effects of free surface parameters on the blast vibration velocity decay were rarely considered, although the free surface parameters affected the blasting effect. Therefore, based on the blast vibration decay prediction formula proposed previously, the blast vibration prediction formula was improved by considering the effects of two parameters, namely, the free surface area and the number of free surfaces, as shown in Eqs. (3) and (4), respectively. The progress of the improved formula was verified using field blast monitoring data. A numerical simulation was performed using LS-DYNA to analyse the decay of the blast vibration velocity under three different operating conditions of tunnel excavation, and the results verified the superiority of the improved formula. The conclusions of this study are as follows:

1. The blast vibration velocity prediction formulas by Sadoff and Lu et al. were improved by introducing two influencing parameters, i.e. the free surface area *S* and the number of surfaces *m*, by incorporating a factor term, (*S*/*mR*^2^)^*β*^. The original blast vibration velocity prediction formula yielded better prediction only under a single operating condition, and the prediction formula can be applied more widely after adding the free surface parameters. The fitting index indicated that the improved prediction formula demonstrated better model selection and fitting.
2. The initial vibration response of the blast vibration was significant; in addition, the amplitude decayed rapidly as the propagation distance increased and then stabilised in the form of an exponential decay.
3. The main frequency range of blast vibration in the initial stage indicated a wide range of frequency bands with clear high-frequency components; as the propagation distance increased, the high-frequency components decayed rapidly, and the frequency band narrowed. The final vibration response was dominated by low frequencies, primarily in the 0–100 Hz range.
